# Recycled Polyethylene/Paraffin Wax/Expanded Graphite Based Heat Absorbers for Thermal Energy Storage: An Artificial Aging Study

**DOI:** 10.3390/molecules24071217

**Published:** 2019-03-28

**Authors:** Haneen Abdelrazeq, Patrik Sobolčiak, Mariam Al-Ali Al-Maadeed, Mabrouk Ouederni, Igor Krupa

**Affiliations:** 1 Department of Chemical Engineering, Qatar University, P.O. Box 2713, Doha, Qatar; ha082881@student.qu.edu.qa; 2 Center for Advanced Materials, Qatar University, P.O. Box 2713, Doha, Qatar; patrik@qu.edu.qa (P.S.); m.alali@qu.edu.qa (M.A.-A.A.-M.); 3 Qatar Petrochemical Company (QAPCO), Doha 756, Qatar; m.ouederni@qapco.com.qa; 4 QAPCO Polymer Chair, Center for Advanced Materials, Qatar University, P.O. Box 2713, Doha, Qatar

**Keywords:** phase change materials, paraffin wax, expanded graphite, artificial aging, leakage, thermal conductivity

## Abstract

An artificial aging study of novel heat absorbers based on phase change materials (PCMs) prepared from recycled high-density polyethylene (HDPE), paraffin wax (PW), and expanded graphite (EG) was investigated. The optimal composition of PCMs contained 40 wt% HDPE, whereas the paraffin wax content ranged from 40 to 60 wt% and the expanded graphite content ranged from 5 to 15 wt%. PCMs were artificially aged through exposure to UV irradiation, enhanced temperature, and humidity. It was clearly demonstrated that the addition of EG to PCMs led to the suppression of PW leakage and improved the photooxidation stability of the PCMs during the aging process. The best performance was achieved by adding 15 wt% of EG to the PCMs. The sample shows a leakage of paraffin wax below 10%, retaining a melting enthalpy of PW within PCMs of 54.8 J/g, a thermal conductivity of 1.64 W/mK and the lowest photooxidation, characterized by an increase in the concentration of carbonyl groups from all investigated materials after artificial aging. Furthermore, PCMs mixed with EG exhibited good mechanical properties, even after 100 days of exposure to artificial aging. Finally, this work demonstrates a justification for the use of recycled plastics in the formation of PCMs.

## 1. Introduction

According to the World Business Council for Sustainable Development, the energy consumption of buildings accounts for approximately 40% of the entire global energy consumed [1]. Today, there is a worldwide trend to utilize renewable sources of energy to address the energetic needs of buildings using local climate conditions. The Sun is the most common source of energy that can be utilized to cover the energetic requirements of buildings. To utilize this energy, various components and materials have been designed and applied in the bioclimatic building concept. These new elements, which can be used for effective absorption and release of thermal energy, contribute to the consumption of electrical energy for heating or cooling.

One category of materials suitable for this purpose is phase change materials (PCMs). They can be characterized as materials that undergo phase transition (from a solid to a liquid and vice versa) at relatively low temperatures while absorbing or releasing a large amount of energy proportional to their specific enthalpy of melting [2,3]. PCMs have gained growing interest in various applications, mostly in the building industry. Various compounds, such as inorganic salts, fatty acids, and paraffin waxes, are widely used as PCMs.

One of the most promising PCMs is paraffin waxes due to their favorable properties, such as high latent heat of fusion, melting temperatures in a broad range, which enables the use of PCMs in outdoor as well as indoor applications, negligible supercooling, chemical stability, and a relatively low price [4,5]. The disadvantage of paraffin waxes is their tendency to leak from the system while in a molten state. One possibility of suppressing the leakage of paraffin wax (PW) from PCMs is based on blending with various polymers [6,7,8]. These materials are called shape-stabilized phase change materials. In this article, for simplicity, materials prepared from the high-density polyethylene (HDPE) matrix, PW and expanded graphite (EG) will be marked as PCMs, although the sole phase change component is PW.

Polyethylene is the most suitable polymer for mixing with paraffin waxes because of its chemical and structural similarities, which make these materials compatible and, in some cases, even miscible with each other [9,10,11]. 

Many scientific papers describing various features of polyethylene/paraffin wax have already been published [12,13,14,15]; however, the major focus has been on virgin polyethylene. Interestingly, there are no significant hindrances to using recycled polyethylene.

The motivation for this approach is the permanent increase in plastic waste accumulation, which requires an exploration of new possibilities for the use of recycled plastics (particularly different grades of polyethylene) for various applications.

One of the possibilities for reusing recycled plastics is the design of recycled plastic/paraffin wax PCMs, as investigated in this paper.

An important feature of organic PCMs is their low thermal conductivity. The thermal conductivity of common plastics ranges from 0.2 to 0.5 W/mK, and the thermal conductivity of paraffin waxes is 0.2 W/mK [16]. An enhancement of the thermal conductivity of PCMs is often required to improve heat transfer between the material and surroundings. Various fillers are employed for that purpose; however, it seems that expanded graphite is the most effective low-cost filler, which also brings additional benefits into the material system, such as an enhancement of mechanical performance and a reduction of PCMs’ flammability [17].

Despite the intensive use of PCMs in indoor and outdoor applications, limited knowledge is available in terms of their long-run performance characterized by changes in mechanical and thermal properties if the materials are exposed to light and higher temperatures. Farid et al. [18] investigated the thermal properties of paraffin wax mixtures and fatty acid mixtures by exposing materials to temperatures beyond their melting point. It was found that the paraffin significantly and irreversibly leaked over time. Fauzi et al. [19] studied the thermal and physical stability of fatty acid mixtures during accelerated melting/solidification processes. It was found that myristic acid/palmitic acid/sodium stearate PCMs have a stable phase transition temperature and latent heat of fusion during 1500 thermal cycles with negligible volume change. In addition, polyethylene glycol-based PCMs were examined via accelerated thermal testing [20]. It was concluded that polyethylene glycol-based PCMs can be effectively used for thermal energy storage for a minimum of five consecutive years, retaining high melting enthalpies.

In this work, the preparation and characterization of a new heat absorber system based on recycled HDPE waste blended with PW and EG was investigated. Prepared PCMs were artificially aged to study their long-term stability, such as the leakage of PW from the PCMs matrix, thermal conductivity, enthalpies of melting and crystallization, and mechanical properties.

## 2. Results and Discussion

### 2.1. Morphology

**Figure 1:**
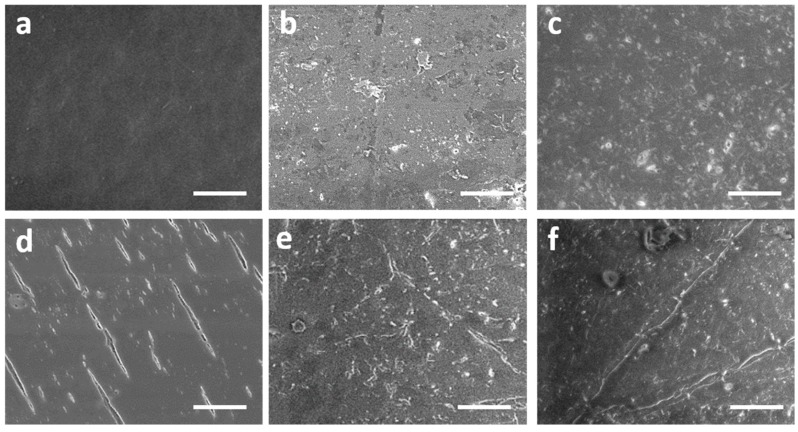
SEM images of the surface of phase change materials (PCMs) before aging—(**a**) S1 as a reference, (**b**) S_3_ and (**c**) S_7_; after aging—(**d**) S_1_, (**e**) S_3_, and (**f**) S_7_. (The bar is equal to 100 µm)

SEM images were captured for pure HDPE, S_3_ and S_7_ before aging (Figure 1a–c) and after one hundred days of aging (Figure 1d–f) to study the outcomes of UV, heat, and humidity during the accelerated aging process.

In the neat HDPE specimen, aging caused a significant introduction of microcracks on the HDPE surface. These cracks are caused by the degradation of polymer chains due to UV irradiation exposure for a prolonged time. This phenomenon is well-known and already described in several studies [21,22]. PCM S_3_ containing 50 wt% PW showed fewer cracks compared to the pure HDPE sample. 

However, PCMs S_7_, which contains the addition of 5 wt% EG, exhibited good mechanical integrity even after aging. The addition of EG to the samples played a very important stabilizing role by reducing the chain mobility and inhibiting degradation in an aging environment [23].

### 2.2. Leakage Analysis

**Figure 2:**
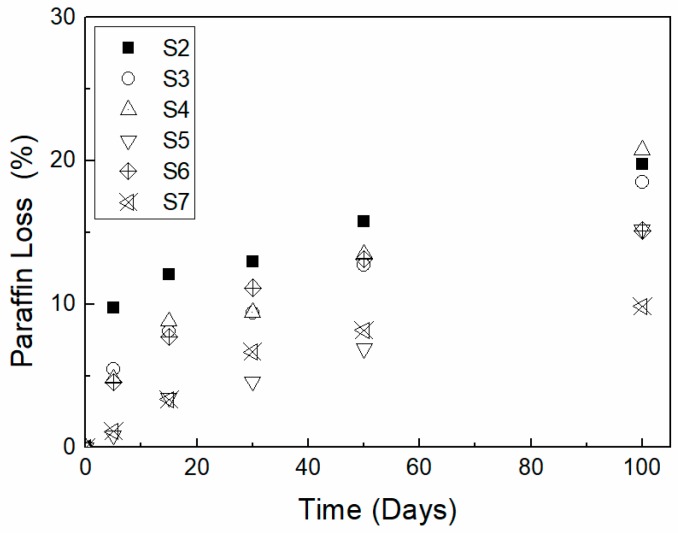
The leakage of PCMs during days of artificial aging.

The leakage of PW from PCMs is a serious issue caused by the mutual immiscibility of PW and HDPE due to their different structures. The highest leakage was observed during 5 days of aging for all studied specimens, most likely due to PW surrounding the surface area of the specimens, which is easily leaked during heating and cooling cycles. During the first 5 days, a noticeable leakage of PW close to the surface was observed (Figure 2).

Based on calculations, the percentages of PW leakage for the PCMs containing 40, 50, and 60 wt% PW were 19, 18, and 20 wt%, respectively. To study the stabilizing effect of EG on the PCMs, a set of samples containing HDPE, 50% PW and various EG contents (from 5 to 15 wt%) were prepared. During artificial aging, different rates of PW leakage for the different PCM compositions were observed. Notably, an increasing concentration of the EG filler caused a decrease in the leakage of PW from the PCMs. Similar observations have been observed previously [24]; these authors studied LLDPE/paraffin wax/expanded graphite composites and found that, among other reasons, the intercalation of EG is caused by the diffusion of PW inside the graphene layers of graphite. The prolonged stability of blends after adding EG is caused by multiple factors, such as an increase in the viscosity of the PCMs, the penetrability of PW chains into the EG graphene sheets, and the suppression of diffusion of the PW through the EG sheet plane.

The lowest PW weight loss (9.83%) was observed for the PCMs containing 15 wt% of EG, whereas PCMs containing 5 wt% and 10 wt% of EG filler showed 15.23% and 15.14% leakage.

### 2.3. FTIR Analysis

The chemical changes of the PCMs during artificial aging were studied by Fourier-transform infrared (FTIR) spectrometry. The appearance and disappearance of peaks corresponding to carbonyl groups (at a wavelength of approximately 1740 cm^−1^) during artificial aging was observed.

**Figure 3:**
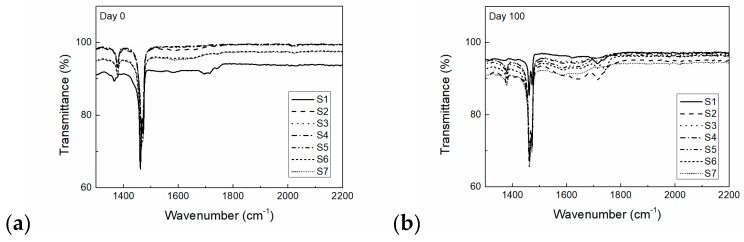
Fourier-transform infrared (FTIR) spectroscopy of PCMs (**a**) before and (**b**) after artificial aging test.

Figure 3 shows the FTIR spectra of the HDPE before and after artificial aging. The FTIR data for the HDPE at the beginning of the aging process contain characteristic peaks at 1463 cm^−1^ due to the -CH_2_ scissoring vibration. In addition, C-C stretching vibration peaks of HDPE appeared at 1031 and 729 cm^−1^ [25].

Several changes in the carbonyl region, such as the increase and broadening of absorption peaks at approximately 1740 cm^−1^ due to aging, were detected. This is an indication of the appearance of multiple oxidation structures [26]. The creation of the C=O band was detected for all studied composites. However, the intensity of a created C=O band was lower compared to pure HDPE.

**Figure 4:**
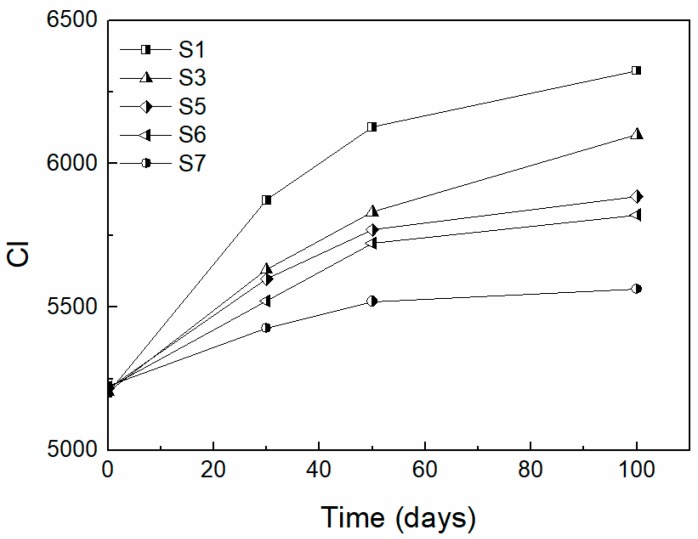
Carbonyl index vs time of aging for high-density polyethylene (HDPE) as a reference, and PCMs with 50 wt% of paraffin wax (PW).

Similarly, enhanced photostability of the PCMs containing EG was observed (Figure 4) through the carbonyl index (CI).

The CI indicates the degree of degradation of HDPE and PW. PW and HDPE are similar in terms of chemical composition; however, PW contains a remarkably lower chain length, which is presumed to have a pronounced effect on the acceleration of the photochemical oxidation of PCMs. The highest increase in CI was observed during the first 30 hours of the aging test for all tested PCMs. An expected decrease of CI for PCMs having 50 wt% of HDPE and 50 wt% of PW compared to pure HDPE was observed. In addition, adding EG caused a decrease in CI due to the photostabilization of the specimens. The CI values significantly decreased with increasing EG content. Similar to other carbon forms (charcoal, CNT), EG restricts photooxidation to the immediate surface sites by preventing both oxygen and light from penetrating into deeper regions of the polymer [27].

The PCMs containing 15 wt% of EG exhibited the best performance in terms of photostability, which is clearly seen in Figure 4, where the CI values were the lowest among all the studied samples.

A similar relation (increased photostability of PCMs due to EG addition) was observed in a previous study [28].

### 2.4. Thermal Investigation

#### 2.4.1. Thermal Conductivity

**Figure 5:**
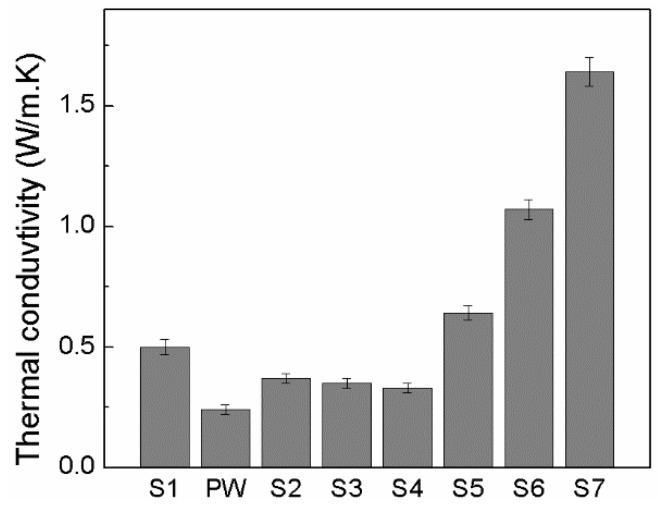
Thermal conductivity of pure components compared to prepared PCMs.

Figure 5 shows a proportional decrease in thermal conductivity as the concentration of PW within the HDPE matrix increased. This expected behavior is caused by differences in the thermal conductivity of PW versus HDPE (0.24 W/mK vs. 0.5 W/mK). The thermal conductivity for all prepared PCMs is shown in Figure 5.

Furthermore, the EG filler significantly enhanced the thermal conductivity of the PCMs due to the much higher conductivity of EG (over 100 W/m·K) compared to the thermal conductivity of PW or HDPE. Additionally, as confirmed by Ling et al. [29], the EG fillers can create a conductive network of PCMs that ensure good heat conduction. The highest thermal conductivity, at 1.64 W/mK, was achieved for the PCM with 15 wt% EG.

#### 2.4.2. Differential Scanning Calorimetry (DSC)

**Figure 6:**
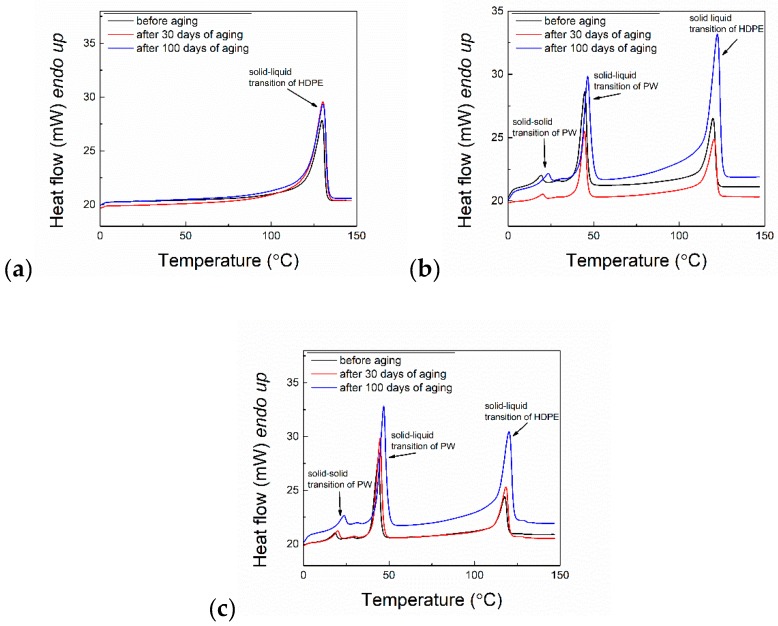
Differential Scanning Calorimetry (DSC) thermographs of (**a**) pure HDPE, (**b**) S_3_ and (**c**) S_7_ PCMs.

The storage and release of thermal heat was examined by DSC measurements for all prepared PCMs. Figure 6 shows the DSC thermographs of selected samples before, after 30 days and after 100 days of artificial aging.

Figure 6a compares the DSC thermographs before, after 30 days, and after 100 days of aging. There are no significant changes in the peak positions or distribution of the peaks, which presumes no significant changes to the crystalline structure of the HDPE chains. The visible peak at approximately 128 °C is due to the solid-liquid transition of HDPE.

Different scenarios can be seen in Figure 6b, where sample S_3_ containing 50 wt% of PW shows a shift of peaks to a higher temperature. The minor peak belongs to the solid-solid transition, which is caused by the reorganizing of the crystalline phase at 22 °C. The aging of the sample caused its shift up to 24.6 °C for the sample measured before aging. The major peak of PW appeared at approximately 44.7 °C and represented melting of the crystalline phase shifted to 46.5 °C due to aging of the PCMs. A slight shift was also observed for the HDPE melting peak at approximately 124 °C. Similarly, a shifting of the peaks was observed for sample S_7,_ as shown in Figure 6c.

It should be noted that the DSC analysis was focused on the thermal investigation of the PW of the PCM blends. The investigation of the effect of PW on the thermal properties of HDPE is beyond the scope of this study. The data, summarized in Table 1 were compiled before and during the running aging test to study the thermal properties (such as temperature and enthalpies of transitions).

The highest decrease of melting enthalpy was observed for the PCMs that did not contain EG. This expected behavior is due to the leakage of PW, as mentioned previously. In the PCMs containing EG (investigation was performed for composites containing 50 wt% of PW), the decrease in melting enthalpies was lower because of the stabilizing effect of EG.

Notably, the noticeable leakage of PW during aging caused the melting enthalpy to decrease. The reduction of melting enthalpy is relative to the PW decrease due to leakage. For instance, PCMs that contain only HDPE and PW exhibited a 38.5% decrease in melting enthalpy after 100 days of aging, whereas the addition of 5 wt% EG caused a slightly suppressed reduction of leakage to 32.4%. Interestingly, after increasing the content of EG to 15 wt%, PCMs exhibited only a 17.2% decrease in melting enthalpy, which is more than double the increased stability of the specimen compared to PCMs without EG. However, to use these PCMs in end-market applications, further strategies to eliminate the leakage of PW from the compact shape of the PCMs need to be implemented.

The best performance in terms of a decrease of enthalpy for melting and crystallization (17.2% and 8.6%, respectively) was shown in PCMs containing 15 wt% EG.

### 2.5. Mechanical Properties of PCMs

The Young’s modulus (E), stress at break (σ), and elongation at break (ε) with the standard deviations (SD) for PCMs are summarized in Table 2.

HDPE, as a very ductile material, has a relatively high E, ε and σ [30]. These properties dramatically changed after adding materials such as PW or EG. First, PW is a soft component with low deformability, causing noticeable decreases in the strength and toughness of HDPE.

The Young’s modulus of pure HDPE was 809 MPa. The addition of PW caused a decrease in the Young’s modulus to 297 MPa for PCM containing 60 wt% of PW due to the significantly lower Young’s modulus of PW versus the modulus of HDPE. A similar trend, a decrease in stress at break of PCMs with increasing PW content, has been observed previously [31].

The elongation at break of pure HDPE was 659%. The addition of PW caused a significant increase in elongation at break to 1285% for PCMs containing 40 wt% of PW due to its plasticizing effect. Further amounts of added PW did not significantly influence the elongation at break. The opposite effect was observed after adding EG. The Young’s modulus of PCMs increased with increasing amounts of EG, which is in line with what we would expect, as the Young’s modulus of graphite is higher than that of HDPE. An increase of Young’s modulus up to 624 MPa for PCMs containing 15 wt% EG was observed. The stress at break of the PCMs increased with EG content up to 5.88 MPa.

The elongation at break of PCMs decreased with loading of EG below 5% for PCMs containing 15 wt% of EG. The remarkable decrease of elongation at break for blends after adding EG is caused by the structure of EG, which consists of many sharp edges, along with the possible structure uniformities of EG particles. All of these structural features of EG decrease the elongation of breaks of composites, as was reported previously [32,33].

## 3. Materials and Methods

### 3.1. Materials

Waste high-density polyethylene (HDPE) with a melting point of approximately 130 °C was supplied in granular form from the Doha Plastic Products & Recycling Co. (Mesaieed Industrial, Doha, Qatar).

A solid paraffin wax of grade RT42 (PW) was used as a PCM material (Rubitherm Technologies, Berlin, Germany). It has a melting temperature of approximately 42 °C.

Expanded graphite (EG) with an average particle size of 200 µm was obtained from SGL Carbon, Germany.

### 3.2. Sample Preparation

The PCMs were manufactured by mixing the required ratio of HDPE, PW, and EG inside a mixing chamber of a Brabender Plasticorder PLE 331 (Duisburg, Germany) for a duration of 10 min at 140 °C and a mixing speed of 35 rpm. The composition of the prepared composites is shown in Table 3.

One-mm thick slabs (60 mm × 40 mm × 1 mm) were prepared by compression molding of the mixed composites using a laboratory press (Fontijne SRA 100, Delft, The Netherlands) at 150 °C for 3 min.

### 3.3. Characterizations

Artificial aging of the PCMs was performed in an accelerated aging tester (Q-Lab, Westlake, OH, USA) according to ISO 4892-3. The temperature of the condensation was 40 °C to ensure the solidification of PW during aging.

Artificial aging involved UV irradiation (model number, company, city, state abbrev. if USA, country) at 340 nm (irradiance 0.76 W/m^2^) at 60 °C for 8 h followed by a condensation step at 40 °C for 4 h. These two steps were repeated for 100 days. PCMs were characterized at the beginning of the aging test and subsequently at certain intervals of artificial aging (Days 5, 15, 30, 50, and 100).

An (FEI Quanta 200, Graz, Austria) environmental scanning electron microscope was used to study the morphology of the prepared PCMs at 2.0 keV at the beginning and after artificial aging. The surfaces of specimens were sputtered with gold to enhance image quality.

Fourier-transform infrared spectroscopy (FTIR) was employed to study the appearance and disappearance of chemical groups on the PCM surface caused by aging. FTIR spectra were obtained on a Spectrum 400 spectrometer (Perkin Elmer, Akron, OH, USA). The absorbance of specimens was measured at wavenumbers (cm^−1^) from 1300 to 2200 cm^−1^. The number of scans for each measurement was 32.

The carbonyl index (CI), which depicts the degree of degradation of the PCMs, was calculated from FTIR spectra. The CI is defined as the peak area of absorbance of the carbonyl band at approximately 1740 cm^−1^ and the internal thickness band at 2020 cm^−1^.

Differential scanning calorimetry (DSC) measurements were performed using a Perkin Elmer model DSC 8500 (Perkin Elmer, Akron, OH, USA) over a temperature range from 0 °C to 150 °C at a heating rate of 10 °C/min under a nitrogen atmosphere. The specific enthalpy of melting (ΔH_m_) was calculated from the second heating curve, whereas the enthalpy of crystallization (ΔH_c_) was calculated from the cooling curve. The nitrogen gas flow rate of the DSC equipment was 40 mL/min. The results obtained from DSC were calculated from three measurements, and average values are presented. The weight of the tested samples varied from 3 to 5 mg.

The leakage of PCMs during aging was calculated to characterize the weight loss of composites from one-mm thick slabs (60 mm × 40 mm × 1 mm) by gravimetric measurement. Specimens were wiped with cotton tissue to remove water and possible PW leakage from the surface of the specimens. The weight loss percentage of all aging samples was calculated according to Equation (1) below: where *m*_0_ is the initial mass of the specimen, *m_x_* is the actual mass of the specimen, and *w* is the mass fraction of paraffin wax.

(1) $$\mathrm{weight}\;\mathrm{loss}\;\left(\%\right)=100-\;\frac{m_{o}-\;m_{x}}{m_{0}\;\times \;w}\;\times 100\%$$

The transient plane source (HotDisk, Goteborg, Sweden) was employed to obtain the thermal conductivity and thermal diffusivity of the composites according to the standard procedure ISO 22007-2. The measurement of thermal conductivity was performed using parallelepiped-shaped samples (45 mm × 45 mm × 5 mm). The TPS sensor was sandwiched between two pieces of specimens. Several tests for each specimen were measured to validate the obtained thermal conductivities of PCMs.

Tensile measurements were performed on dog-bone-shaped specimens with a working area of 30 mm × 4 mm × 1 mm, which were cut from the slabs. The mechanical properties were measured using an Instron 3365 (TestResources Inc., Shakopee, MN, USA) universal testing machine in tensile mode at room temperature. The deformation rate was 10 mm·min^−1^. Seven tensile measurements were performed per sample. The average values were obtained from performing at least seven tensile tests per sample composition.

## 4. Conclusions

Phase change materials with the ability to be used as novel heat absorbers were prepared from recycled high-density polyethylene, paraffin wax and expanded graphite. Materials underwent an artificial aging process at selected temperatures, UV irradiation and humidity up to 100 days, and the changes in their thermophysical and mechanical properties were evaluated. A strong effect of aging was observed, especially for PCMs without the addition of EG. The samples containing EG showed good mechanical integrity even after 100 days of aging, whereas those without EG cracked after 10 to 20 days of aging. The PCMs were also able to absorb and release thermal energy up to 77.4 J/g in the case of PCM with 60 wt% PW. It was found that EG has a complex, positive influence on the long-term stability of PCMs with respect to (i) an improvement of thermal conductivity, (ii) the suppression of PW leakage from compact PCMs, and (iii) a prolongation of their photostability.

## Figures and Tables

**Table 1:** DSC melting temperatures and enthalpies for the different compositions of PCMs during aging.

HDPE/PW/EG	60/40		50/50		40/60		45/50/5		40/50/10		35/50/15	
Time (Days)	T_m_ (°C)	ΔH_m_ (J/g)	T_m_ (°C)	ΔH_m_ (J/g)	T_m_ (°C)	ΔH_m_ (J/g)	T_m_ (°C)	ΔH_m_ (J/g)	T_m_ (°C)	ΔH_m_ (J/g)	T_m_ (°C)	ΔH_m_ (J/g)
0	43.8 (0.3)	47.0 (1.3)	44.7 (0.2)	61.8 (2.2)	44.3 (0.2)	77.4 (2.0)	44.4 (0.3)	63.3 (3.2)	43.9 (0.3)	63.2 (3.8)	43.9 (0.3)	66.2 (3.7)
5	44.3 (0.2)	43.5 (2.2)	44.8 (0.3)	60.2 (3.2)	45.5 (0.3)	71.5 (4.2)	44.0 (0.5)	61.5 (3.1)	44.4 (0.3)	61.6 (3.3)	45.1 (0.6)	61.9 (2.7)
15	45.1 (0.4)	32.4 (2.8)	44.2 (0.3)	53 (2.8)	46.0 (0.5)	62.3 (4.1)	44.6 (0.3)	56.9 (2.0)	43.9 (0.1)	58.1 (3.8)	43.8 (0.2)	60.2 (3.5)
30	45.9 (0.2)	36.0 (3.2)	44.6 (0.4)	53.9 (1.2)	45.6 (0.3)	50.7 (4.2)	44.5 (0.4)	58 (2.7)	44.5 (0.3)	57.7 (4.0)	44.8 (0.3)	56.8 (3.0)
50	45.3 (0.3)	30.8 (2.7)	45.1 (0.3)	48.3 (4.2)	46.0 (0.6)	55.7 (4.0)	44.8 (0.4)	51.6 (4.0)	44.5 (0.5)	55.5 (2.9)	45.5 (0.3)	55.2 (5.0)
100	45.9 (0.3)	26.0 (2.5)	46.5 (0.2)	38 (2.9)	47.2 (0.5)	42.5 (4.1)	46.2 (0.6)	42.8 (3.5)	44.8 (0.5)	50.2 (2.8)	44.8 (0.5)	54.8 (4.5)
%Drop in ΔH_m_		44.7%		38.5%		45.1%		32.4%		20.6%		17.2%

**Table 2:** Mechanical properties of PCMs obtained from tensile measurements.

Sample HDPE/PW/EG	Young’s Modulus		Stress at Break		Elongation at Break	
	E (MPa)	SD	σ (MPa)	SD	ε (%)	SD
100-0-0	809	34	20.3	6.6	659	288
60-40-0	419	33	13.7	1.4	1285	144
50-50-0	356	25	9.14	0.72	1203	114
40-60-0	297	36	6.08	0.26	1109	95
45-50-5	435	19	4.53	0.68	15.3	4.9
40-50-10	518	54	5.82	0.26	7.96	1.78
35-50-15	624	46	5.88	0.32	4.63	0.39

**Table 3:** Composition of prepared samples.

Sample ID	HDPE wt%	PW wt%	EG wt%
S1	100	0	0
S2	60	40	0
S3	50	50	0
S4	40	60	0
S5	45	50	5
S6	40	50	10
S7	35	50	15

## Abbreviations

CI	carbonyl index
DSC	Differential Scanning Calorimetry
FTIR	Fourier-transform infrared spectroscopy
HDPE	high-density polyethylene
E	Young’s modulus
EG	expanded graphite
*m*_0_	initial mass of a specimen
*m_x_*	actual mass of a specimen
keV	kilo electron volt
PCMs	phase change materials
PE	polyethylene
PP	polypropylene
PS	polystyrene
PW	paraffin wax
SEM	Scanning Electron Microscopy
SD	standard deviation
T_m_	melting temperature
TPS	transient plane source
UV	ultraviolet
w	mass fraction of paraffin wax
W	watt
wt%	weight percentage
ΔH_m_	enthalpy of melting
σ	stress at break and
ε	elongation at break
